# Benzene-1,3-diammonium bis­(pyridine-2,6-dicarboxyl­ato-κ^3^ *O* ^2^,*N*,*O* ^6^)cobaltate(II) penta­hydrate

**DOI:** 10.1107/S1600536811009858

**Published:** 2011-03-31

**Authors:** Hoda Pasdar, Saghi Sadat Kashani, Reza Ghiasi, Hossein Aghabozorg, Behrouz Notash

**Affiliations:** a Department of Chemistry, Islamic Azad University, North Tehran Branch, Tehran, Iran; b Department of Chemistry, Basic Science Faculty, East Tehran Branch, Islamic Azad University, Qiam Dasht, Tehran, Iran; c Department of Chemistry, Shahid Beheshti University, G. C., Evin, Tehran 1983963113, Iran

## Abstract

In the title compound, (C_6_H_10_N_2_)[Co(C_7_H_3_NO_4_)_2_]·5H_2_O, the Co^II^ ion is six-coordinated in an N_2_O_4_ environment by two pyridine-2,6-dicarboxyl­ate (pydc) ligands, having a distorted octa­hedral geometry. The crystal packing is stabilized by inter­molecular N—H⋯O, O—H⋯O and weak C—H⋯O hydrogen bonds. There are also π–π inter­actions between the pyridine rings of the pydc ligands and between the pydc ligands and the benzene-1,3-diammonium cations, with centroid–centroid distances of 3.4575 (15) and 3.7521 (15) Å.

## Related literature

For general background to proton-transfer compounds, see: Aghabozorg *et al.* (2008 ▶). For related structures, see: Beatty *et al.* (2002 ▶); Dobrzycki & Woźniak (2008 ▶); Imaz *et al.* (2007 ▶); Pasdar *et al.* (2010 ▶, 2011*a* ▶,*b* ▶).
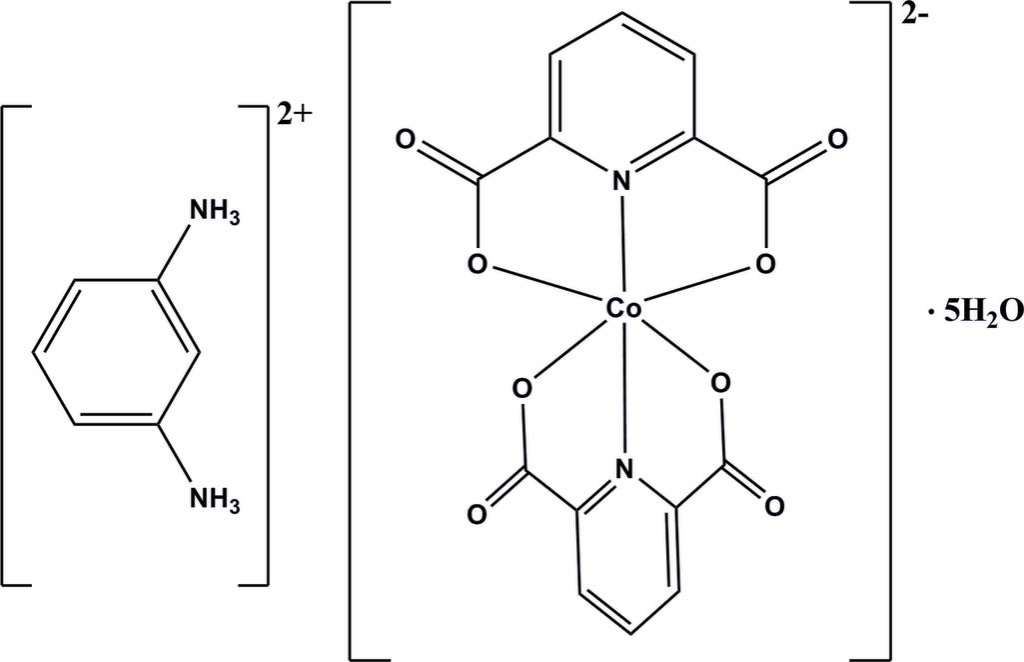


## Experimental

### 

#### Crystal data

(C_6_H_10_N_2_)[Co(C_7_H_3_NO_4_)_2_]·5H_2_O
*M* *_r_* = 589.38
Monoclinic, 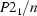

*a* = 7.5236 (2) Å
*b* = 18.0200 (7) Å
*c* = 18.7122 (6) Å
β = 100.883 (2)°
*V* = 2491.29 (14) Å^3^
*Z* = 4
Mo *K*α radiation
μ = 0.76 mm^−1^
*T* = 298 K
0.50 × 0.15 × 0.10 mm

#### Data collection

Stoe IPDS-2 diffractometer
19874 measured reflections
6702 independent reflections
5366 reflections with *I* > 2σ(*I*)
*R*_int_ = 0.064

#### Refinement

*R*[*F* ^2^ > 2σ(*F* ^2^)] = 0.055
*wR*(*F* ^2^) = 0.113
*S* = 1.21
6702 reflections
407 parameters
2 restraints
H atoms treated by a mixture of independent and constrained refinement
Δρ_max_ = 0.34 e Å^−3^
Δρ_min_ = −0.32 e Å^−3^

### 

Data collection: *X-AREA* (Stoe & Cie, 2005 ▶); cell refinement: *X-AREA*; data reduction: *X-AREA*; program(s) used to solve structure: *SHELXS97* (Sheldrick, 2008 ▶); program(s) used to refine structure: *SHELXL97* (Sheldrick, 2008 ▶); molecular graphics: *ORTEP-3* (Farrugia, 1997 ▶); software used to prepare material for publication: *WinGX* (Farrugia, 1999 ▶).

## Supplementary Material

Crystal structure: contains datablocks I, global. DOI: 10.1107/S1600536811009858/hy2407sup1.cif

Structure factors: contains datablocks I. DOI: 10.1107/S1600536811009858/hy2407Isup2.hkl

Additional supplementary materials:  crystallographic information; 3D view; checkCIF report

## Figures and Tables

**Table 1:** Hydrogen-bond geometry (Å, °)

*D*—H⋯*A*	*D*—H	H⋯*A*	*D*⋯*A*	*D*—H⋯*A*
C10—H10⋯O3^i^	0.93	2.57	3.311 (3)	136
C18—H18⋯O8^ii^	0.93	2.47	3.099 (3)	125
O9—H9*A*⋯O3	0.86 (4)	1.97 (4)	2.789 (3)	160 (3)
O9—H9*B*⋯O10	0.76 (3)	2.07 (3)	2.833 (4)	176 (4)
O10—H10*A*⋯O6^i^	0.81 (6)	2.10 (6)	2.913 (4)	173 (5)
O10—H10*B*⋯O11	0.80 (5)	1.97 (5)	2.764 (5)	170 (5)
O11—H11*A*⋯O8	0.97 (5)	1.84 (5)	2.746 (4)	153 (4)
O11—H11*B*⋯O13^iii^	0.86 (5)	2.08 (5)	2.907 (4)	161 (5)
O12—H12*A*⋯O10	0.93 (7)	2.03 (7)	2.946 (5)	171 (5)
O12—H12*B*⋯O2^iv^	0.73 (5)	2.09 (5)	2.786 (4)	161 (5)
O13—H13*A*⋯O12	0.86 (3)	1.95 (3)	2.805 (4)	176 (5)
O13—H13*B*⋯O5^v^	0.78 (5)	2.13 (5)	2.873 (3)	161 (5)
N3—H3*A*⋯O4^iii^	0.87 (4)	1.93 (4)	2.791 (3)	169 (3)
N3—H3*B*⋯O7	0.96 (4)	1.78 (4)	2.714 (3)	163 (3)
N3—H3*C*⋯O13^iii^	0.98 (4)	2.04 (4)	2.890 (4)	144 (3)
N3—H3*C*⋯O9^iii^	0.98 (4)	2.29 (4)	2.899 (3)	120 (3)
N4—H4*A*⋯O9	0.89 (4)	1.97 (4)	2.844 (3)	168 (4)
N4—H4*B*⋯O2^iv^	0.90 (4)	1.87 (4)	2.752 (3)	166 (3)
N4—H4*C*⋯O6^v^	0.88 (4)	2.00 (4)	2.873 (3)	175 (3)

Symmetry codes: (i) 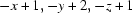; (ii) 
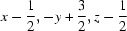
; (iii) 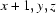; (iv) 
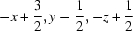
; (v) 
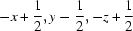
.

**Fig. 1.**
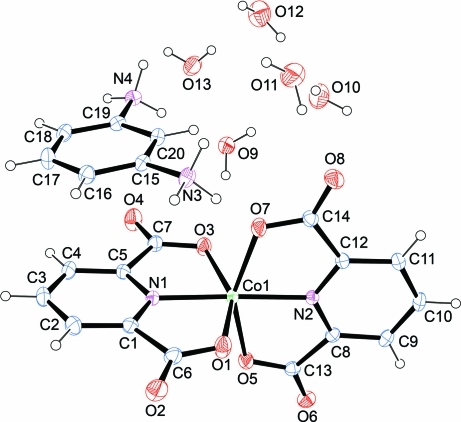
The molecular structure of the title compound, with displacement ellipsoids drawn at the 30% probability level.

**Fig. 2.**
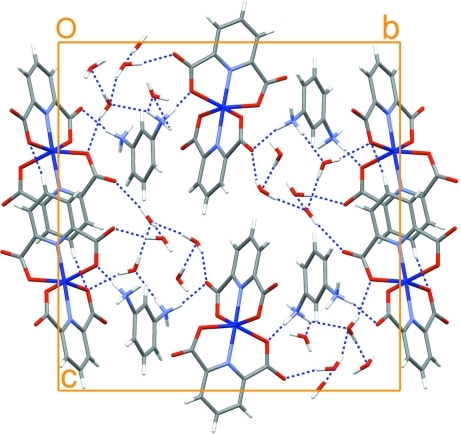
The packing diagram of the title compound, viewed down the a axis. Intermolecular N—H···O, O—H···O and weak C—H···O hydrogen bonds are shown as blue dashed lines.

**Fig. 3.**
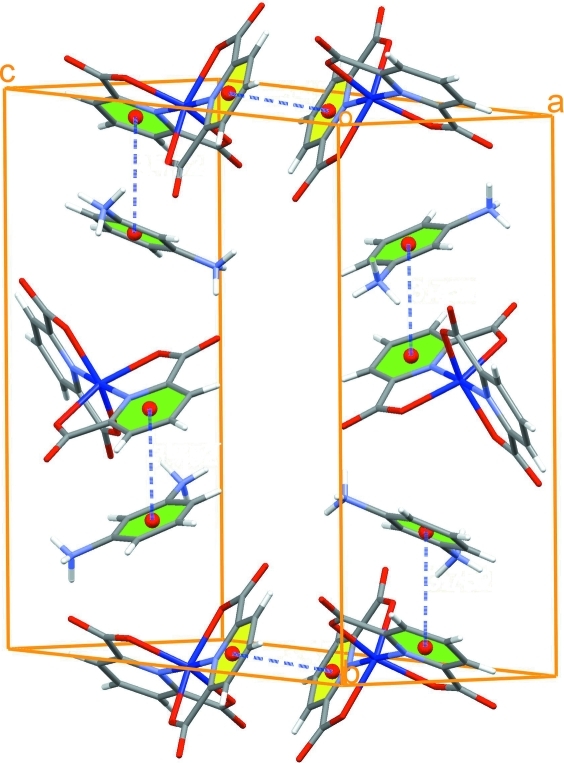
The packing diagram of the title compound, showing intermolecular π–π interactions (dashed lines) between the pyridine rings of the pydc ligands [centroid–centroid distance = 3.4575 (15) Å] and between the benzene rings of the (bdaH2)2+ cations and the pyridine rings of the pydc ligands [centroid–centroid distance = 3.7521 (15) Å]. Water molecules have been omitted for clarity.

## supplementary crystallographic information

### Comment

Our group used pyridine-2,6-dicarboxylic acid (pydcH_2_) in several proton-transfer systems (Aghabozorg *et al.*, 2008; Pasdar *et al.*, 2010, 2011*a*,*b*). Benzene-1,3-diamine (bda) has ability to act as a proton acceptor in proton-transfer compounds. The formation of mono- (Beatty *et al.*, 2002) and diprotonated benzene-1,3-diamine (Dobrzycki & Woźniak, 2008; Imaz *et al.*, 2007) has been observed previously.

Herein, we report the synthesis and crystal structure of the title compound (Fig. 1). The Co^II^ ion in the complex anion is six-coordinated by two tridentate pydc ligands in a distorted octahedral geometry. We have recently reported the synthesis and crystal structure of a nickle(II) analogue, (bdaH_2_)[Ni(pydc)_2_].5H_2_O (Pasdar *et al.*, 2011*b*). The nickle(II) and cobalt(II) complexes are isostructural. Crystal packing of the title compound is stabilized by intermolecular N—H···O, O—H···O and weak C—H···O hydrogen bonds (Fig. 2, Table 1). There are also π–π interactions between the pyridine rings of the pydc ligands and between the pyridine ring of the pydc ligand and the benzene ring of the benzene-1,3-diammonium cation, with centroid–centroid distances of 3.4575 (15) and 3.7521 (15) Å.

### Experimental

A solution of pydcH_2_ (162 mg, 0.9 mmol) in 17 ml water was added to a solution of bda (108 mg, 0.6 mmol) in 11 ml water with stirring for an hour, and then a solution of CoCl_2_.6H_2_O (118 mg, 0.6 mmol) in 5 ml water was added. The resulting solution was stirred for 2 h and dark pink crystals of the title compound suitable for X-ray analysis were obtained after one week (m.p. 260°C).

### Refinement

H atoms of water molecules and N—H groups of (bdaH_2_)^2+^ cation were found in a difference Fourier map and refined isotropically. H9B and H13A were refined with distance restraints of O—H = 0.76 (3) and 0.86 (3) Å. H atoms of the aromatic rings were positioned geometrically and refined as riding atoms, with C—H = 0.93 Å and *U*_iso_(H) = 1.2*U*_eq_(C).

### Crystal data

**Table:**

(C_6_H_10_N_2_)[Co(C_7_H_3_NO_4_)_2_]·5H_2_O	*F*(000) = 1220
*M**_r_* = 589.38	*D*_x_ = 1.571 Mg m^−^^3^
Monoclinic, *P*2_1_/*n*	Mo *K*α radiation, λ = 0.71073 Å
Hall symbol: -P 2yn	Cell parameters from 6702 reflections
*a* = 7.5236 (2) Å	θ = 2.2–29.2°
*b* = 18.0200 (7) Å	µ = 0.76 mm^−^^1^
*c* = 18.7122 (6) Å	*T* = 298 K
β = 100.883 (2)°	Needle, dark pink
*V* = 2491.29 (14) Å^3^	0.50 × 0.15 × 0.10 mm
*Z* = 4	

### Data collection

**Table:**

Stoe IPDS-2 diffractometer	5366 reflections with *I* > 2σ(*I*)
Radiation source: fine-focus sealed tube	*R*_int_ = 0.064
graphite	θ_max_ = 29.2°, θ_min_ = 2.2°
ω scans	*h* = −10→9
19874 measured reflections	*k* = −23→24
6702 independent reflections	*l* = −25→25

### Refinement

**Table:**

Refinement on *F*^2^	Primary atom site location: structure-invariant direct methods
Least-squares matrix: full	Secondary atom site location: difference Fourier map
*R*[*F*^2^ > 2σ(*F*^2^)] = 0.055	Hydrogen site location: inferred from neighbouring sites
*wR*(*F*^2^) = 0.113	H atoms treated by a mixture of independent and constrained refinement
*S* = 1.21	*w* = 1/[σ^2^(*F*_o_^2^) + (0.031*P*)^2^ + 1.7282*P*] where *P* = (*F*_o_^2^ + 2*F*_c_^2^)/3
6702 reflections	(Δ/σ)_max_ = 0.007
407 parameters	Δρ_max_ = 0.34 e Å^−^^3^
2 restraints	Δρ_min_ = −0.32 e Å^−^^3^

### Fractional atomic coordinates and isotropic or equivalent isotropic displacement parameters (Å^2^)

**Table:**

	*x*	*y*	*z*	*U*_iso_*/*U*_eq_	
Co1	0.64449 (5)	0.982592 (19)	0.312577 (16)	0.02479 (9)	
O1	0.8779 (3)	1.03503 (13)	0.28454 (10)	0.0426 (5)	
O2	1.0026 (3)	1.06589 (13)	0.19026 (12)	0.0481 (6)	
O3	0.4033 (3)	0.91494 (11)	0.28828 (9)	0.0341 (4)	
O4	0.2123 (3)	0.86302 (14)	0.19579 (11)	0.0471 (5)	
O5	0.4980 (3)	1.08443 (11)	0.31941 (10)	0.0355 (4)	
O6	0.4177 (3)	1.16437 (10)	0.39852 (11)	0.0348 (4)	
O7	0.8000 (3)	0.88934 (11)	0.35988 (9)	0.0367 (4)	
O8	0.9307 (3)	0.83948 (12)	0.46576 (11)	0.0448 (5)	
O9	0.3372 (3)	0.77720 (13)	0.34605 (11)	0.0393 (5)	
O10	0.5258 (5)	0.72787 (18)	0.48332 (16)	0.0614 (7)	
O11	0.8434 (5)	0.69320 (18)	0.43693 (16)	0.0659 (7)	
O12	0.3223 (4)	0.5943 (2)	0.42544 (17)	0.0670 (8)	
O13	0.0351 (4)	0.65086 (15)	0.32207 (15)	0.0519 (6)	
N1	0.6128 (3)	0.96490 (11)	0.20453 (10)	0.0251 (4)	
N2	0.6870 (3)	1.00350 (10)	0.42057 (10)	0.0220 (4)	
N3	0.9659 (3)	0.80230 (14)	0.27389 (12)	0.0292 (4)	
N4	0.3688 (3)	0.68621 (13)	0.22490 (13)	0.0282 (4)	
C1	0.7387 (4)	0.99011 (14)	0.16927 (13)	0.0288 (5)	
C2	0.7267 (4)	0.97588 (17)	0.09568 (14)	0.0392 (6)	
H2	0.8153	0.9927	0.0711	0.047*	
C3	0.5791 (4)	0.93595 (17)	0.05974 (14)	0.0404 (7)	
H3	0.5683	0.9257	0.0104	0.048*	
C4	0.4476 (4)	0.91124 (16)	0.09695 (13)	0.0356 (6)	
H4	0.3470	0.8851	0.0731	0.043*	
C5	0.4702 (3)	0.92658 (14)	0.17044 (12)	0.0261 (5)	
C6	0.8870 (4)	1.03362 (15)	0.21823 (14)	0.0327 (5)	
C7	0.3483 (3)	0.89943 (14)	0.22113 (13)	0.0290 (5)	
C8	0.6188 (3)	1.06555 (13)	0.44373 (12)	0.0242 (4)	
C9	0.6530 (4)	1.08338 (15)	0.51711 (13)	0.0301 (5)	
H9	0.6071	1.1268	0.5334	0.036*	
C10	0.7571 (4)	1.03510 (15)	0.56585 (13)	0.0309 (5)	
H10	0.7816	1.0462	0.6153	0.037*	
C11	0.8246 (3)	0.97036 (14)	0.54083 (12)	0.0266 (5)	
H11	0.8943	0.9375	0.5728	0.032*	
C12	0.7848 (3)	0.95620 (13)	0.46633 (12)	0.0228 (4)	
C13	0.5029 (3)	1.10953 (13)	0.38304 (13)	0.0261 (5)	
C14	0.8452 (3)	0.88883 (14)	0.42920 (13)	0.0277 (5)	
C15	0.8107 (3)	0.77539 (13)	0.22120 (12)	0.0242 (4)	
C16	0.8140 (4)	0.77813 (15)	0.14768 (13)	0.0304 (5)	
H16	0.9120	0.7987	0.1310	0.036*	
C17	0.6676 (4)	0.74947 (17)	0.09941 (13)	0.0359 (6)	
H17	0.6678	0.7504	0.0497	0.043*	
C18	0.5211 (4)	0.71944 (15)	0.12409 (13)	0.0312 (5)	
H18	0.4236	0.7002	0.0913	0.037*	
C19	0.5212 (3)	0.71837 (13)	0.19752 (13)	0.0250 (5)	
C20	0.6661 (3)	0.74587 (14)	0.24743 (12)	0.0266 (5)	
H20	0.6659	0.7445	0.2971	0.032*	
H3A	1.041 (5)	0.8267 (19)	0.2526 (18)	0.042 (9)*	
H4A	0.342 (5)	0.714 (2)	0.261 (2)	0.057 (11)*	
H9A	0.381 (5)	0.819 (2)	0.3366 (18)	0.043 (9)*	
H10A	0.543 (7)	0.755 (3)	0.519 (3)	0.093 (18)*	
H11A	0.903 (7)	0.741 (3)	0.439 (3)	0.087 (16)*	
H12A	0.387 (9)	0.634 (4)	0.448 (3)	0.12 (2)*	
H13A	0.125 (6)	0.633 (3)	0.352 (3)	0.11 (2)*	
H3B	0.929 (5)	0.8373 (19)	0.3069 (18)	0.044 (9)*	
H4B	0.403 (5)	0.642 (2)	0.2465 (19)	0.052 (10)*	
H9B	0.385 (5)	0.763 (2)	0.3831 (17)	0.055 (12)*	
H10B	0.624 (7)	0.719 (3)	0.475 (2)	0.068 (15)*	
H11B	0.886 (7)	0.671 (3)	0.403 (3)	0.081 (15)*	
H12B	0.380 (7)	0.580 (3)	0.401 (3)	0.074 (16)*	
H13B	0.033 (7)	0.624 (3)	0.289 (3)	0.089 (17)*	
H3C	1.035 (5)	0.762 (2)	0.301 (2)	0.057 (11)*	
H4C	0.277 (5)	0.6805 (19)	0.1891 (19)	0.045 (9)*	

### Atomic displacement parameters (Å^2^)

**Table:**

	*U*^11^	*U*^22^	*U*^33^	*U*^12^	*U*^13^	*U*^23^
Co1	0.02959 (17)	0.02648 (16)	0.01790 (13)	−0.00151 (14)	0.00348 (11)	−0.00162 (12)
O1	0.0403 (11)	0.0565 (14)	0.0311 (9)	−0.0213 (10)	0.0069 (8)	−0.0076 (9)
O2	0.0504 (13)	0.0507 (13)	0.0487 (12)	−0.0263 (11)	0.0232 (10)	−0.0087 (10)
O3	0.0383 (10)	0.0402 (10)	0.0258 (8)	−0.0118 (8)	0.0109 (7)	−0.0033 (7)
O4	0.0383 (11)	0.0640 (15)	0.0388 (10)	−0.0237 (10)	0.0065 (9)	−0.0065 (10)
O5	0.0451 (11)	0.0319 (10)	0.0269 (8)	0.0085 (8)	0.0001 (8)	0.0007 (7)
O6	0.0304 (10)	0.0303 (10)	0.0425 (10)	0.0070 (8)	0.0039 (8)	−0.0020 (8)
O7	0.0486 (12)	0.0358 (10)	0.0244 (8)	0.0155 (9)	0.0036 (8)	−0.0048 (7)
O8	0.0575 (14)	0.0389 (11)	0.0366 (10)	0.0198 (10)	0.0052 (9)	0.0045 (9)
O9	0.0457 (12)	0.0370 (12)	0.0338 (10)	−0.0053 (9)	0.0035 (9)	−0.0008 (9)
O10	0.0624 (19)	0.0697 (19)	0.0494 (14)	0.0069 (15)	0.0035 (13)	−0.0104 (13)
O11	0.082 (2)	0.0566 (17)	0.0617 (17)	0.0052 (15)	0.0205 (15)	−0.0063 (14)
O12	0.0659 (19)	0.084 (2)	0.0579 (16)	0.0049 (17)	0.0296 (15)	−0.0025 (15)
O13	0.0587 (16)	0.0464 (14)	0.0483 (13)	0.0015 (11)	0.0047 (12)	−0.0124 (11)
N1	0.0312 (11)	0.0235 (10)	0.0206 (8)	−0.0037 (8)	0.0054 (7)	−0.0010 (7)
N2	0.0222 (9)	0.0237 (10)	0.0203 (8)	−0.0007 (7)	0.0045 (7)	−0.0012 (7)
N3	0.0210 (10)	0.0362 (12)	0.0301 (10)	−0.0029 (9)	0.0036 (8)	−0.0084 (9)
N4	0.0239 (10)	0.0273 (11)	0.0331 (11)	−0.0015 (8)	0.0048 (9)	−0.0008 (9)
C1	0.0346 (13)	0.0261 (12)	0.0271 (11)	−0.0048 (10)	0.0093 (9)	0.0001 (9)
C2	0.0521 (17)	0.0404 (15)	0.0294 (12)	−0.0096 (13)	0.0184 (11)	−0.0011 (11)
C3	0.0572 (19)	0.0425 (16)	0.0224 (11)	−0.0083 (13)	0.0099 (11)	−0.0042 (11)
C4	0.0434 (15)	0.0367 (14)	0.0247 (11)	−0.0083 (12)	0.0009 (10)	−0.0068 (10)
C5	0.0276 (12)	0.0261 (12)	0.0242 (10)	−0.0022 (9)	0.0036 (9)	0.0001 (9)
C6	0.0352 (13)	0.0302 (13)	0.0346 (12)	−0.0081 (10)	0.0118 (10)	−0.0034 (10)
C7	0.0279 (12)	0.0311 (13)	0.0282 (11)	−0.0048 (10)	0.0058 (9)	−0.0014 (10)
C8	0.0227 (11)	0.0241 (11)	0.0263 (10)	−0.0014 (8)	0.0056 (8)	−0.0023 (9)
C9	0.0322 (13)	0.0304 (12)	0.0285 (11)	−0.0010 (10)	0.0077 (10)	−0.0102 (10)
C10	0.0337 (13)	0.0384 (14)	0.0204 (10)	−0.0044 (10)	0.0044 (9)	−0.0079 (9)
C11	0.0249 (11)	0.0335 (13)	0.0212 (10)	−0.0012 (9)	0.0036 (8)	0.0022 (9)
C12	0.0205 (10)	0.0271 (11)	0.0213 (10)	0.0000 (8)	0.0053 (8)	0.0002 (8)
C13	0.0223 (11)	0.0249 (11)	0.0307 (11)	−0.0024 (9)	0.0036 (9)	−0.0003 (9)
C14	0.0277 (12)	0.0284 (12)	0.0266 (11)	0.0023 (9)	0.0043 (9)	0.0008 (9)
C15	0.0194 (10)	0.0261 (11)	0.0263 (10)	0.0018 (9)	0.0022 (8)	−0.0058 (9)
C16	0.0287 (12)	0.0345 (13)	0.0295 (12)	−0.0015 (10)	0.0094 (10)	−0.0021 (10)
C17	0.0377 (15)	0.0482 (16)	0.0219 (11)	−0.0040 (12)	0.0060 (10)	−0.0036 (11)
C18	0.0298 (13)	0.0331 (13)	0.0284 (11)	−0.0040 (10)	−0.0004 (9)	−0.0074 (10)
C19	0.0200 (11)	0.0238 (11)	0.0312 (11)	0.0007 (9)	0.0044 (9)	−0.0027 (9)
C20	0.0252 (12)	0.0310 (12)	0.0235 (10)	−0.0004 (9)	0.0042 (9)	−0.0035 (9)

### Geometric parameters (Å, °)

**Table:**

Co1—N1	2.0160 (19)	N4—C19	1.461 (3)
Co1—N2	2.0209 (18)	N4—H4A	0.89 (4)
Co1—O7	2.1414 (19)	N4—H4B	0.90 (4)
Co1—O1	2.145 (2)	N4—H4C	0.88 (4)
Co1—O5	2.1570 (19)	C1—C2	1.387 (3)
Co1—O3	2.1621 (19)	C1—C6	1.521 (4)
O1—C6	1.256 (3)	C2—C3	1.386 (4)
O2—C6	1.241 (3)	C2—H2	0.9300
O3—C7	1.277 (3)	C3—C4	1.387 (4)
O4—C7	1.233 (3)	C3—H3	0.9300
O5—C13	1.268 (3)	C4—C5	1.381 (3)
O6—C13	1.242 (3)	C4—H4	0.9300
O7—C14	1.277 (3)	C5—C7	1.519 (3)
O8—C14	1.228 (3)	C8—C9	1.386 (3)
O9—H9A	0.86 (4)	C8—C13	1.518 (3)
O9—H9B	0.76 (3)	C9—C10	1.390 (4)
O10—H10A	0.81 (6)	C9—H9	0.9300
O10—H10B	0.80 (5)	C10—C11	1.389 (4)
O11—H11A	0.97 (5)	C10—H10	0.9300
O11—H11B	0.86 (5)	C11—C12	1.393 (3)
O12—H12A	0.93 (7)	C11—H11	0.9300
O12—H12B	0.73 (5)	C12—C14	1.511 (3)
O13—H13A	0.86 (3)	C15—C16	1.381 (3)
O13—H13B	0.78 (5)	C15—C20	1.382 (3)
N1—C5	1.332 (3)	C16—C17	1.386 (4)
N1—C1	1.333 (3)	C16—H16	0.9300
N2—C12	1.328 (3)	C17—C18	1.383 (4)
N2—C8	1.336 (3)	C17—H17	0.9300
N3—C15	1.462 (3)	C18—C19	1.374 (3)
N3—H3A	0.87 (4)	C18—H18	0.9300
N3—H3B	0.96 (4)	C19—C20	1.386 (3)
N3—H3C	0.98 (4)	C20—H20	0.9300
			
N1—Co1—N2	177.16 (8)	C5—C4—C3	118.0 (2)
N1—Co1—O7	104.03 (7)	C5—C4—H4	121.0
N2—Co1—O7	76.23 (7)	C3—C4—H4	121.0
N1—Co1—O1	76.81 (8)	N1—C5—C4	121.1 (2)
N2—Co1—O1	100.37 (7)	N1—C5—C7	113.2 (2)
O7—Co1—O1	92.18 (9)	C4—C5—C7	125.6 (2)
N1—Co1—O5	103.23 (8)	O2—C6—O1	125.5 (3)
N2—Co1—O5	76.60 (7)	O2—C6—C1	118.7 (2)
O7—Co1—O5	152.70 (7)	O1—C6—C1	115.8 (2)
O1—Co1—O5	95.22 (9)	O4—C7—O3	125.9 (2)
N1—Co1—O3	76.36 (7)	O4—C7—C5	119.1 (2)
N2—Co1—O3	106.48 (7)	O3—C7—C5	115.0 (2)
O7—Co1—O3	90.92 (8)	N2—C8—C9	120.3 (2)
O1—Co1—O3	152.93 (7)	N2—C8—C13	113.31 (19)
O5—Co1—O3	94.25 (8)	C9—C8—C13	126.4 (2)
C6—O1—Co1	115.64 (17)	C8—C9—C10	118.7 (2)
C7—O3—Co1	115.67 (16)	C8—C9—H9	120.6
C13—O5—Co1	115.76 (16)	C10—C9—H9	120.6
C14—O7—Co1	116.43 (16)	C11—C10—C9	120.1 (2)
H9A—O9—H9B	111 (4)	C11—C10—H10	120.0
H10A—O10—H10B	106 (5)	C9—C10—H10	120.0
H11A—O11—H11B	102 (4)	C10—C11—C12	117.9 (2)
H12A—O12—H12B	104 (5)	C10—C11—H11	121.0
H13A—O13—H13B	100 (5)	C12—C11—H11	121.0
C5—N1—C1	121.5 (2)	N2—C12—C11	121.1 (2)
C5—N1—Co1	119.62 (16)	N2—C12—C14	113.42 (19)
C1—N1—Co1	118.82 (16)	C11—C12—C14	125.5 (2)
C12—N2—C8	121.87 (19)	O6—C13—O5	125.3 (2)
C12—N2—Co1	119.28 (15)	O6—C13—C8	119.3 (2)
C8—N2—Co1	118.84 (15)	O5—C13—C8	115.3 (2)
C15—N3—H3A	111 (2)	O8—C14—O7	125.6 (2)
C15—N3—H3B	111 (2)	O8—C14—C12	119.8 (2)
H3A—N3—H3B	105 (3)	O7—C14—C12	114.6 (2)
C15—N3—H3C	112 (2)	C16—C15—C20	122.1 (2)
H3A—N3—H3C	107 (3)	C16—C15—N3	119.8 (2)
H3B—N3—H3C	110 (3)	C20—C15—N3	118.1 (2)
C19—N4—H4A	110 (3)	C15—C16—C17	118.3 (2)
C19—N4—H4B	109 (2)	C15—C16—H16	120.8
H4A—N4—H4B	104 (3)	C17—C16—H16	120.8
C19—N4—H4C	110 (2)	C18—C17—C16	120.9 (2)
H4A—N4—H4C	112 (3)	C18—C17—H17	119.5
H4B—N4—H4C	111 (3)	C16—C17—H17	119.5
N1—C1—C2	120.6 (2)	C19—C18—C17	119.3 (2)
N1—C1—C6	112.8 (2)	C19—C18—H18	120.4
C2—C1—C6	126.6 (2)	C17—C18—H18	120.4
C3—C2—C1	118.3 (2)	C18—C19—C20	121.4 (2)
C3—C2—H2	120.9	C18—C19—N4	120.3 (2)
C1—C2—H2	120.9	C20—C19—N4	118.3 (2)
C2—C3—C4	120.4 (2)	C15—C20—C19	118.0 (2)
C2—C3—H3	119.8	C15—C20—H20	121.0
C4—C3—H3	119.8	C19—C20—H20	121.0
			
N1—Co1—O1—C6	2.1 (2)	C3—C4—C5—C7	−175.3 (3)
N2—Co1—O1—C6	−177.6 (2)	Co1—O1—C6—O2	173.7 (2)
O7—Co1—O1—C6	106.0 (2)	Co1—O1—C6—C1	−4.3 (3)
O5—Co1—O1—C6	−100.3 (2)	N1—C1—C6—O2	−173.2 (3)
O3—Co1—O1—C6	9.7 (3)	C2—C1—C6—O2	6.8 (4)
N1—Co1—O3—C7	−3.76 (19)	N1—C1—C6—O1	5.0 (4)
N2—Co1—O3—C7	176.12 (18)	C2—C1—C6—O1	−174.9 (3)
O7—Co1—O3—C7	−107.98 (19)	Co1—O3—C7—O4	−178.5 (2)
O1—Co1—O3—C7	−11.4 (3)	Co1—O3—C7—C5	4.3 (3)
O5—Co1—O3—C7	98.85 (19)	N1—C5—C7—O4	−179.7 (3)
N1—Co1—O5—C13	−177.44 (18)	C4—C5—C7—O4	−3.2 (4)
N2—Co1—O5—C13	−0.35 (18)	N1—C5—C7—O3	−2.3 (3)
O7—Co1—O5—C13	5.3 (3)	C4—C5—C7—O3	174.2 (3)
O1—Co1—O5—C13	−99.79 (19)	C12—N2—C8—C9	1.8 (3)
O3—Co1—O5—C13	105.59 (19)	Co1—N2—C8—C9	−176.81 (18)
N1—Co1—O7—C14	177.36 (19)	C12—N2—C8—C13	−176.8 (2)
N2—Co1—O7—C14	0.26 (19)	Co1—N2—C8—C13	4.6 (3)
O1—Co1—O7—C14	100.4 (2)	N2—C8—C9—C10	−0.9 (4)
O5—Co1—O7—C14	−5.4 (3)	C13—C8—C9—C10	177.6 (2)
O3—Co1—O7—C14	−106.5 (2)	C8—C9—C10—C11	−0.1 (4)
O7—Co1—N1—C5	89.93 (19)	C9—C10—C11—C12	0.1 (4)
O1—Co1—N1—C5	178.9 (2)	C8—N2—C12—C11	−1.7 (3)
O5—Co1—N1—C5	−88.78 (19)	Co1—N2—C12—C11	176.86 (17)
O3—Co1—N1—C5	2.43 (18)	C8—N2—C12—C14	178.6 (2)
O7—Co1—N1—C1	−88.0 (2)	Co1—N2—C12—C14	−2.8 (3)
O1—Co1—N1—C1	0.90 (19)	C10—C11—C12—N2	0.7 (4)
O5—Co1—N1—C1	93.24 (19)	C10—C11—C12—C14	−179.7 (2)
O3—Co1—N1—C1	−175.5 (2)	Co1—O5—C13—O6	−174.9 (2)
O7—Co1—N2—C12	1.51 (17)	Co1—O5—C13—C8	2.7 (3)
O1—Co1—N2—C12	−88.20 (18)	N2—C8—C13—O6	173.0 (2)
O5—Co1—N2—C12	178.85 (19)	C9—C8—C13—O6	−5.5 (4)
O3—Co1—N2—C12	88.34 (18)	N2—C8—C13—O5	−4.8 (3)
O7—Co1—N2—C8	−179.87 (19)	C9—C8—C13—O5	176.7 (2)
O1—Co1—N2—C8	90.42 (18)	Co1—O7—C14—O8	178.4 (2)
O5—Co1—N2—C8	−2.53 (17)	Co1—O7—C14—C12	−1.7 (3)
O3—Co1—N2—C8	−93.04 (18)	N2—C12—C14—O8	−177.2 (2)
C5—N1—C1—C2	−1.2 (4)	C11—C12—C14—O8	3.1 (4)
Co1—N1—C1—C2	176.7 (2)	N2—C12—C14—O7	2.9 (3)
C5—N1—C1—C6	178.8 (2)	C11—C12—C14—O7	−176.7 (2)
Co1—N1—C1—C6	−3.2 (3)	C20—C15—C16—C17	−0.7 (4)
N1—C1—C2—C3	1.0 (4)	N3—C15—C16—C17	177.5 (2)
C6—C1—C2—C3	−179.0 (3)	C15—C16—C17—C18	0.5 (4)
C1—C2—C3—C4	0.1 (5)	C16—C17—C18—C19	0.2 (4)
C2—C3—C4—C5	−1.1 (5)	C17—C18—C19—C20	−0.9 (4)
C1—N1—C5—C4	0.2 (4)	C17—C18—C19—N4	−179.3 (2)
Co1—N1—C5—C4	−177.8 (2)	C16—C15—C20—C19	0.1 (4)
C1—N1—C5—C7	176.9 (2)	N3—C15—C20—C19	−178.1 (2)
Co1—N1—C5—C7	−1.0 (3)	C18—C19—C20—C15	0.7 (4)
C3—C4—C5—N1	1.0 (4)	N4—C19—C20—C15	179.2 (2)

### Hydrogen-bond geometry (Å, °)

**Table:**

*D*—H···*A*	*D*—H	H···*A*	*D*···*A*	*D*—H···*A*
C10—H10···O3^i^	0.93	2.57	3.311 (3)	136
C18—H18···O8^ii^	0.93	2.47	3.099 (3)	125
O9—H9A···O3	0.86 (4)	1.97 (4)	2.789 (3)	160 (3)
O9—H9B···O10	0.76 (3)	2.07 (3)	2.833 (4)	176 (4)
O10—H10A···O6^i^	0.81 (6)	2.10 (6)	2.913 (4)	173 (5)
O10—H10B···O11	0.80 (5)	1.97 (5)	2.764 (5)	170 (5)
O11—H11A···O8	0.97 (5)	1.84 (5)	2.746 (4)	153 (4)
O11—H11B···O13^iii^	0.86 (5)	2.08 (5)	2.907 (4)	161 (5)
O12—H12A···O10	0.93 (7)	2.03 (7)	2.946 (5)	171 (5)
O12—H12B···O2^iv^	0.73 (5)	2.09 (5)	2.786 (4)	161 (5)
O13—H13A···O12	0.86 (3)	1.95 (3)	2.805 (4)	176 (5)
O13—H13B···O5^v^	0.78 (5)	2.13 (5)	2.873 (3)	161 (5)
N3—H3A···O4^iii^	0.87 (4)	1.93 (4)	2.791 (3)	169 (3)
N3—H3B···O7	0.96 (4)	1.78 (4)	2.714 (3)	163 (3)
N3—H3C···O13^iii^	0.98 (4)	2.04 (4)	2.890 (4)	144 (3)
N3—H3C···O9^iii^	0.98 (4)	2.29 (4)	2.899 (3)	120 (3)
N4—H4A···O9	0.89 (4)	1.97 (4)	2.844 (3)	168 (4)
N4—H4B···O2^iv^	0.90 (4)	1.87 (4)	2.752 (3)	166 (3)
N4—H4C···O6^v^	0.88 (4)	2.00 (4)	2.873 (3)	175 (3)

Symmetry codes: (i) −*x*+1, −*y*+2, −*z*+1; (ii) *x*−1/2, −*y*+3/2, *z*−1/2; (iii) *x*+1, *y*, *z*; (iv) −*x*+3/2, *y*−1/2, −*z*+1/2; (v) −*x*+1/2, *y*−1/2, −*z*+1/2.
